# Betulinic and ursolic acids from *Nauclea latifolia* roots mediate their antimalarial activities through docking with PfEMP-1 and PfPKG proteins

**DOI:** 10.1186/s12906-023-04324-x

**Published:** 2024-02-07

**Authors:** Edet Effiong Asanga, Ndifreke Daniel Ekpo, Affiong Asuquo Edeke, Chinedum Martins Ekeleme, Henshaw Uchechi Okoroiwu, Uwem Okon Edet, Ekementeabasi A. Umoh, Nikita Elkanah Umoaffia, Olorunfemi Abraham Eseyin, Ani Nkang, Monday Akpanabiatu, Jude Efiom Okokon, Sylvia Akpotuzor, Bright Asuquo Effiong, MacGeorge Ettaba

**Affiliations:** 1 Department of Biochemistry, Arthur Jarvis University, Akpabuyo, Cross River State Nigeria; 2 Department of Biochemistry, University of Uyo, Uyo, Akwa Ibom State Nigeria; 3 Department of Biochemistry, TopFaith University, Mkpatak, Akwa Ibom State Nigeria; 4 Department of Medical Laboratory Science, David Umahi Federal University of Health Sciences, Uburu, Ebonyi State Nigeria; 5 Department of Microbiology, Arthur Jarvis University, Akpabuyo, Cross River State Nigeria; 6 Department of Human Physiology, Arthur Jarvis University, Akpabuyo, Cross River State Nigeria; 7 Department of Medicinal and Pharmaceutical Chemistry, University of Uyo, Uyo, Akwa Ibom State Nigeria; 8 Department of Biological Sciences, Arthur Jarvis University, Akpabuyo, Cross River State Nigeria; 9 Department of Biochemistry, AkwaIbom State University, IkotAkpaden, Mkpatenin, Akwa Ibom State Nigeria; 10 Department of Pharmacology and Toxicology, University of Uyo, Uyo, Akwa Ibom State Nigeria; 11 Department of Mathematics and Computer Science, Arthur Jarvis University, Akpabuyo, Cross River State Nigeria

**Keywords:** *Nauclea latifolia*, Malaria, Betulinic acid, Ursolic acid, *Plasmodium berghei*, Docking, GCMS

## Abstract

**Background:**

Chemotherapies target the PfEMP-1 and PfPKG proteins in *Plasmodium falciparum*, the parasite that causes malaria, in an effort to prevent the disease’s high fatality rate. This work identified the phytochemical components of *Nauclea latifolia* roots and docked the chemical compounds against target proteins, and examined the in vivo antiplasmodial effect of the roots on *Plasmodium berghei*-infected mice.

**Methods:**

Standard protocols were followed for the collection of the plant’s roots, cleaning, and drying of the roots, extraction and fraction preparation, assessment of the in vivo antiplasmodial activity, retrieval of the PfEMP-1 and PfPKG proteins, GCMS, ADME, and docking studies, chromatographic techniques were employed to separate the residual fraction’s components, and the Swis-ADME program made it possible to estimate the drug’s likeness and pharmacokinetic properties. The Auto Dock Vina 4.2 tool was utilized for molecular docking analysis.

**Results:**

The residual fraction showed the best therapeutic response when compared favorably to amodiaquine (80.5%) and artesunate (85.1%). It also considerably reduced the number of parasites, with the % growth inhibition of the parasite at 42.8% (D2) and 83.4% (D5). Following purification, 25 compounds were isolated and characterized with GCMS. Based on their low molecular weights, non-permeation of the blood–brain barrier, non-inhibition of metabolizing enzymes, and non-violation of Lipinski’s criteria, betulinic and ursolic acids were superior to chloroquine as the best phytochemicals. Hence, they are lead compounds.

**Conclusion:**

In addition to identifying the bioactive compounds, ADME, and docking data of the lead compounds as candidates for rational drug design processes as observed against *Plasmodium falciparum* target proteins (PfEMP-1 and PfPKG), which are implicated in the pathogenesis of malaria, the study has validated that the residual fraction of *N. latifolia* roots has the best antiplasmodial therapeutic index.

## Background

Malaria is a lethal illness brought about by *Plasmodium falciparum* that is spread by female Anopheles mosquitoes. *Plasmodium falciparum* enhances serious pathogenesis and, if not properly treated, can lead to death [1]. They are found in the red blood cells and liver cells of the individual; however, it is believed that *Plasmodium falciparum* erythrocyte membrane protein 1 (PfEMP-1) binds to the endothelial cell receptors CD-36, thrombospondin (TSP), and intercellular adhesion molecule 1 (ICAM-1) and that there is a receptor related to malaria that mediates the attachment of pRBCs to the microvascular endothelium. The specific binding point on PfEMP-1 and corresponding receptors on the host cells could potentially serve as targets for creating substances aimed at reversing the binding of pRBCs, thereby stopping the blockage of micro-vessels and offering a promising approach for treating malaria [2].

In addition, some researchers have suggested a potential mechanism involving cGMP binding, which could offer valuable insights for advancing biochemical investigations. They provided direct preclinical validation of *Plasmodium falciparum* protein kinase G (PfPKG) as a target for treatment against malaria [3], and it was documented that a PfPKG inhibitor derived from imidazopyridine effectively eliminated *P. falciparum* infection in mice that had been transplanted with human red blood cells. Moreover, they proposed the principle of “chemoprotection” for the intended anti-malarial product profile. This concept entails combining a number of medications that are effective against hepatic schizonts of Plasmodium and may also be active against asexual blood stages [4]. Their results offered proof of concept that it is possible to inhibit the pre-erythrocytic cycle by specifically and selectively chemically targeting Plasmodium PKG, making it a target for the creation of chemo-protective medications.

Ethnopharmacologically, the roots of *Nauclea latifolia* are utilized for treating malaria [5]. This plant is categorized within the Rubiaceae family and is employed to address various health issues, including toothaches, malaria, diarrhea, and dysentery [6]. The bark of the plant yielded five monoterpeneindole alkaloids, naucleamides A to E, with naucleamide E being the most abundant, with an amino-acetal bridge and five cyclic ring configurations [7]. Additionally, betulinic acid, a pentacycliclupane-type triterpenoid that is naturally occurring, has been separated from different plant parts and has been shown to possess antiplasmodial activity [8, 9], as well as ursolic acids, which have been reported to possess anti-inflammatory, anticancer, antimalarial, antioxidant, and antidiabetic activities [10].

Moreover, despite the practice of managing malaria with traditional medicine and chemotherapies, the validation of the mode of action of the lead compounds is a serious concern. Therefore, the discovery of therapeutics has raised concern on how ligands are docked with receptors. In fact, it defines the ligands’ insertion into their protein-binding pockets as well as the interactions that influence the ideal placement of the protein–ligand complexes. It also identifies the different intermolecular interactions that work best for defining the characteristics and strength of the binding affinity. In the early stages of drug discovery, researchers profile many natural products to develop lead compounds that may lead to increased efficacy. Hence, the initial phase of a substantial drug discovery and development initiative involves lead identification. During this process, chemical compounds are pinpointed that interact with the protein and alter its function. In order to be effective against target proteins like *Plasmodium falciparum* cGMP-dependent protein kinase (PfPKG) and *Plasmodium falciparum* erythrocyte membrane proteins-1 (PfEMP-1), lead chemicals should ideally exhibit some degree of precision and potency.

This study was geared towards the validation of the antiplasmodial potentials of *Nauclea latifolia* roots, the isolation and characterization of its bioactive compounds, as well as their in silico prediction against PfPKG and PfEMP-1 proteins.

## Methods

### Preparation of plant material, extraction, and isolation

In January 2021, the roots of *Nauclea latifolia* were harvested in Ikot Andem Ididep, Ibiono Ibom L.G.A., Akwa Ibom State, Nigeria. The roots were identified by Mr. Etefia Umoh, a plant taxonomist at the University of Uyo in Nigeria’s Department of Pharmacognosy and Natural Medicine. The voucher specimen was deposited, and herbarium number UUH67G was recorded. Permission was given to use an identified name (as this is the default in the faculty). The roots were then chopped into pieces and chunks, rinsed under flowing tap water, drained of water, dried under shade for days, and then ground. The 5.2 kg of ground roots were macerated in 8 L of boiled water for 72 h and shaken intermittently to obtain the aqueous extract of the roots. Filtration was done by pouring the mixture into a filter paper; afterwards, the filtrate was concentrated using a rotary evaporator at 40 °C*.*

The aqueous extract had a weight and yield of 246.9 g and 4.75%, respectively. The extract (150 g) was successively partitioned with ethyl acetate (28 × 250 ml) and butanol (42 × 250 ml) using a separating funnel (1000 ml), and the results were the ethyl acetate fraction (13.66 g), butanol fraction (52.83 g), and residual fraction (83.51 g). On the extract and fractions, tests for acute toxicity and antiplasmodial activity were conducted. The residual fraction had the best results against *Plasmodium berghei* in the in vivo antiplasmodial study. The residual fraction (10 g) was subjected to a purification protocol through the silica gel in column chromatography (CC), and the appropriate solvent systems (n-hexane (600 ml), dichloromethane (1.4 L), ethyl acetate (1.9 L), and methanol (2.4 L) were used to elute the residual fraction. This procedure produced 358 fractions. Each of these fractions passed through two distinct TLC analysis phases (the pooling process and then the bulking process), and as a result, twenty-four (24) and six (6) fractions (A–G) were produced based on similar Rf values. Following CC using silica gel, bulked fraction C (3428 mg) was eluted using the appropriate solvent mixtures to yield 47 sub-fractions. Six bulked sub-fractions (C1–C6) were obtained after these sub-fractions were subjected to TLC. Analytical pre-coated TLC silica gel 60 from Sigma-Aldrich was used for thin-layer chromatography, and the TLC plates were then viewed under both long (366 nm) and short (254 nm) wavelength UV light. In addition, the silica gel Kiesel gel 60 (200–400 mesh, Merck) and Sephadex LH-20 from Sigma-Aldrich improved column chromatography (CC) analysis.

### Animals

The Animal House in the Faculty of Pharmacy, University of Uyo, Nigeria, provided fifty-four (54) albino mice (13–27 g) used for the antiplasmodial experiment. The NIH protocols for the use, handling, and care of laboratory animals were judiciously followed [11]. Throughout the experiment, the mice had access to water, a regular pelleted feed, and housing at room temperature. The experimental protocol was strictly followed and authorized by the faculty of Pharmaceutical Science animal ethics committee (approval number: UUFPHARM/0317). The study was carried out in accordance with ARRIVE guidelines for reporting animal experiments.

### Parasites

The donor mice infected with chloroquine-sensitive *P. berghei* (NK-65) utilized in the study were donated by the National Institute of Medical Research (NIMR), Nigeria.

### Preparation of the inoculum

A collection of *Plasmodium berghei*-infected red blood cells was obtained, featuring a minimal peripheral parasitemia level of 20%. This collection was obtained through the cardiac puncture of the infected mice and was gathered in a tube with an anticoagulant. By comparing the count of *Plasmodium berghei-*infected erythrocytes to the count of leukocytes, the percentage of parasitemia was calculated. To create a serial dilution, normal saline was utilized, and the final inoculum (0.2 ml) comprised approximately 1 × 10^7^*Plasmodium berghei*-infected erythrocytes, considered the established standard inoculum for mice [5].

### Drugs

Both artesunate (50 mg) and amodiaquine (200 mg) tablets, manufactured by Mekophar Chemical Pharmaceutical Joint-stock Company in Ho Chi Minh City, Vietnam, were dissolved in 100 ml of distilled water. These drugs were administered at levels of 5 mg/kg and 30 mg/kg, respectively, in the antiplasmodial experiment as positive controls [5].

### Assessment of the plant extract’s LD50

The LD50 and effective dosages of the plant’s root extracts were calculated using Lorke’s method [12]. After administering the aqueous extract, ethyl acetate, butanol, and residual fractions (50–2000 mg/kg) through the peritoneum, toxicity symptoms such as gasping, slowed breathing, palpitations, and death could be seen within 24 h.

### The measurement of in vivo antimalarial activity

As described by Asanga et al*.* [5], the antiplasmodial potentials of the extract, ethyl acetate, butanol, and residual fractions were assessed utilizing the curative model previously reported as the Rane’s test model and modified by Asanga et al. [5]. Parasite density was deduced by using the formula:

$$\mathrm{No}.\;\mathrm{of}\;\mathrm{parasites}\;\mathrm{counted}\;\times\:8000/\mathrm{WBC}\;\mathrm{Counts}=\mathrm{Parasites}/{\mu}\mathrm{L}.$$

The mean survival time (MST) of each treatment group was determined over a period of 30 days (D_0_-D_29_) as the sum of survival times for each group and the number of mice in that group.

Percentage (%) growth inhibition was calculated as:

$$\frac{\mathrm{Control}\;(\mathrm{baseline}\;\mathrm{parasite}\;\mathrm{density}\;\mathrm{on}\;{\mathrm D}_0)\;\mathrm{-parasite}\;\mathrm{densities}\;\mathrm{on}\;({\mathrm D}_2\;\mathrm{or}\;{\mathrm D}_5)\:\times\:100}{\mathrm{Control}\;(\mathrm{baseline}\;\mathrm{parasite}\;\mathrm{density}\;\mathrm{on}\;{\mathrm D}_{0})\;}$$

### Slide staining and examination

Blood was drawn from each mouse and used to create two types of smears: thin and thick. However, there were minor adjustments made to the experimental procedure, which were outlined in a previous report by Owusu-Agyei et al. [13]. The parasite densities per microliter (µL) of blood were determined by counting against 200 and 500 leukocytes.

### Gas chromatography – mass spectroscopy of partially purified compounds

Each of the isolates—C1, C5, and F—was weighed at 10 mg and then dissolved in dimethyl sulfoxide (DMSO). Two (2) µL (split ratio 10:1; split flow 12 mL/min) were introduced into an Agilent system made up of a model 7890N gas chromatograph and a model Triple Quad 7000A mass detector using electron ionization (EI) at 70 eV (mass-to-charge ratio range: 40–600 amu; Agilent Technologies, Santa Clara, California, USA). The gas chromatography (GC) column utilized was an HP-5 ms fused silica capillary equipped with a 5% phenyl-methyl polysiloxane stationary phase (30 m × 250 μm × 0.25 μm). Helium was employed as the carrier gas, with a column head pressure of 9.7853 psi and a flow rate of 1.2 mL/min. The inlet temperature was set at 250 °C, while the temperature of the mass selective detector was maintained at 250 °C. The GC oven temperature program was as follows: the initial temperature of 50 °C was held for 10 min, then increased at a rate of 6 °C/min to 190 °C for 20 min, and further increased at a rate of 7 °C/min to 290 °C for 30 min. The National Institute of Standards and Technology (NIST) database and ChemStation data system were used to compare the retention indices and mass spectrum fragmentation patterns of the isolates to identify the compounds inside them. The GC–MS instrument is housed at the Dr. Panjwani Centre for Molecular Medicine and Drug Research, International Centre for Chemical and Biological Sciences, University of Karachi, Karachi, Pakistan.

### The assessment of the physicochemical and pharmacokinetic properties of the bioactive compounds from *Nauclea latifolia* roots

The drug-likeness and ADME (Absorption, Distribution, Metabolism, and Excretion) properties of the bioactive compounds were assessed using established methods as detailed in a prior publication [14]. The canonical strings, or Simplified Molecular-Input Line-Entry System (SMILES), representations of the various compounds were retrieved from the PubChem web platform (https://www.ncbi.nlm.nih.gov/pccompound) in their 3D conformation. All relevant parameters, including Lipinski’s Rule of Five, Egan, Muegge, Veber, and the Ghose parameters, are presented in Table 6.

### Target protein properties and molecular docking of the bioactive compounds

The protein–ligand interaction in this study was determined through the use of the Auto Dock Vina 4.2 tool, while Biovia Discovery Studio software was used for visualization of the interaction. Two target proteins, *Plasmodium falciparum* erythrocyte membrane protein 1 (PfEMP-1) and cGMP-dependent protein kinase (PKG) (PfPKG), with PDB ID codes 7JGD and 5DYL, were downloaded in PDB format from the Research Collaboratory for Structural Bioinformatics (RCSB) protein data bank. These proteins were prepared for interaction using the Biovia Discovery Studio software by removing water molecules, heteroatoms, and ligands. After the preparation, the proteins were blindly docked against the studied bioactive compounds. For comparison, chloroquine, an antimalarial drug, was obtained from the drug bank and docked; their binding affinities and hydrogen bonds were documented. The target protein grid parameters were as follows: 7JGD: Center X = 156.911000, Center Y = 160.262000, and Center Z = 141.132000, as well as 5DYL: Center X = -38.312000, Center Y = 40.579000, and Center Z = 28.371000, respectively.

### Analysis of statistics

Data analysis was done using GraphPad Prism version 8.0 (GraphPad Software, Inc., San Diego, CA, USA). The results were presented using the mean and standard error of the mean (SEM), and a one-way ANOVA was used to statistically analyze any significant variations between and among the groups. Following that, Turkey’s post hoc test improved multiple means comparisons, and differences were determined to be statistically significant at a 95% confidence level.

## Results

### The schizonticidal activity of extract and fractions

This study compared the negative control, amodiaquine, and artesunate groups with parasite densities, mean survival times, and percentage growth inhibition of *Plasmodium berghei* to determine the schizonticidal potentials of the extract, ethyl acetate, butanol and residual fractions of *Nauclea latifolia* root at dosages of 150, 300, and 450 mg/kg. Table 1 showed that, at a 95% confidence level, the parasite densities on days two (D2) and five (D5) were significantly reduced in each group compared with the corresponding negative control group. All treatment groups, with the exception of the ethyl acetate fraction, revealed a statistically significant increase in MST in comparison with the group acting as the negative control, according to a 95% confidence level.

**Table 1:** In vivo antiplasmodial activity (schizonticidal test model) (*n* = 6) group

Treatment group	Parasite density/µL on D_0 &_ % growth inhibition	Parasite density/µL on D_2 &_ % growth inhibition	Parasite density/µL on D_5 &_ % growth inhibition	Mean survival time (days)
Negative control (distilled water)	134,021.0 ± 15,652.0	177,052.0 ± 28,312.0 (-32.2%)	222,876.1 ± 30,656.1 (-66.4%)^*^	9.6 ± 0.24
Amodiaquine (30 mg/kg)	130,995.1 ± 16,454.1	62,746.0 ± 6998.0^a*^ (52.2%)^**^	25,516.0 ± 2292.0^a*^ (80.8%)^**^	22.6 ± 0.23^a**^
Artesunate (5 mg/kg)	169,896.1 ± 12,742.1	90,092.0 ± 3098.0^a*^ (47.1%)	24,004.1 ± 3745.1^a*^ (85.7%)^**^	24.2 ± 1.33^a**^
Aqueous extract (150 mg/kg)	204,132.1 ± 10,805.1 (3.4%)	99,256.0 ± 2273.0^a*^ (51.4%)^**^	57,679.1 ± 494.6^a*^ (71.7%)^**^	19.66 ± 0.57^a**^
Aqueous extract (300 mg/kg)	241,429.1 ± 40,596	88,112.0 ± 14,842.0^a*^ (63.4%)^**^	54,017.2 ± 17,178.1^a*^ (77.8%)^**^	22.2 ± 0.43^a**^
Aqueous extract (450 mg/kg)	236,963.1 ± 51,136.1	118,903.0 ± 13,298.0 (49.9%)	60,522.0 ± 14,058.1^a*^ (74.6%)^**^	20.4 ± 0.36^a**^
Ethyl acetate fraction (300 mg/kg)	168,675.1 ± 18,613.1	145,654.0 ± 24,429.0^b**^ (13.7%)	89,443.2 ± 9388.1^a*^ (47.2%)	15.3 ± 0.36^b*, c*^
Butanol fraction (300 mg/kg)	171,646.1 ± 8238.6	120,106.0 ± 21,748.0 (30.3%)	61,678.1 ± 11,616.1^a*^ (64.2%)^**^	17.1 ± 0.42^a**, c*^
Residual fraction (300 mg/kg)	206,217.1 ± 43,858.1	117,922.0 ± 16,843.0 (42.8%)	46,138.1 ± 5267.0^a*^ (83.4%%)^**^	22.2 ± 0.46^a**^

^**^ Significant increases; *P* < 0.05 versus baseline parasite density on day 1 (D_0_); a*significant decreases; *P* < 0.05 versus negative control; a** implies significant increases *P* < 0.05 versus negative control; b* implies significant decreases *P* < 0.05 versus amodiaquine; b** implies significant increases *P* < 0.05 versus amodiaquine; c* implies significant decreases *P* < 0.05 versusartesunate

### GCMS profiling of the sub-fractions of *Nauclea latifolia* roots

**Fig. 1:**
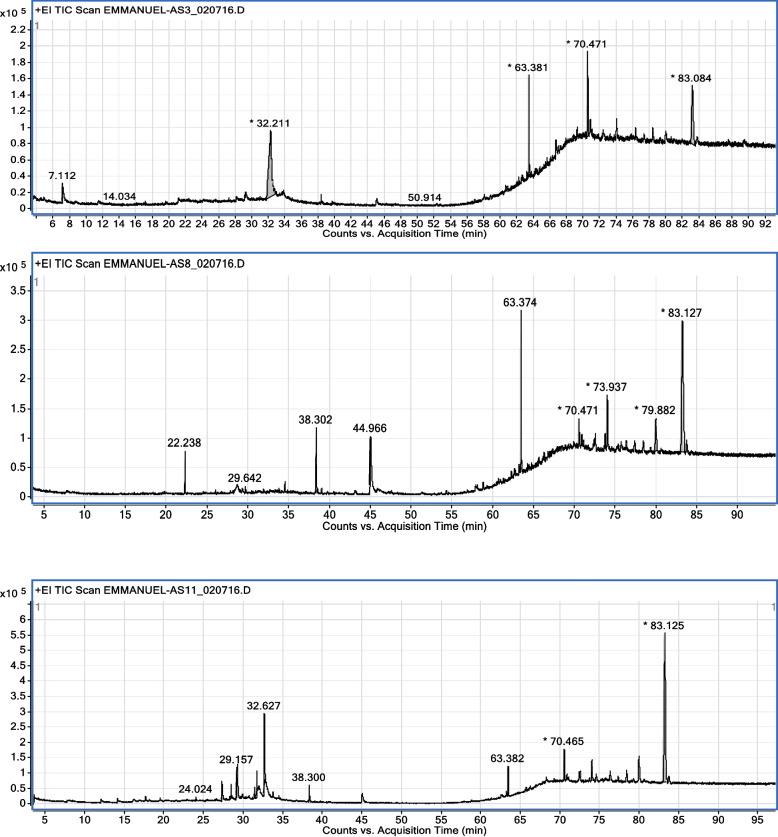
Chromatogram of semi-purified compounds AS3 (C_1_), AS8 (C_5_), and AS11 (F)

The spectroscopic analysis (Fig. 1 and Tables 2, 3 and 4) of the various sub-fractions obtained from the chromatographic analysis revealed the molecular formula, molecular weight, and retention time for the following bioactive compounds: Spiro(androst-5-ene-17,1'-cyclobutan) -2'-one, 3-hydroxy (A), 3-O-methyl-d-glucose (B), 1,2-benzene dicarboxylic acid, diisooctylester (C), 9,10-secocholesta-5,7,10(19) -triene-3,24,25 triol (D), Urs-12-en-28-oic,3-hydroxy-methylester (ursolic acid) (E), 1,2-benzene dicarboxylic acid, mono(2-ethyl hexyl)ester (F), 1,2-benzene dicarboxylic acid, bis (2-methyl propyl)ester (G), 7,9-Di-tert-butyl-1-oxaspiro(4,5) deca-6,9-diene-2,8-dione (I), n-heptadecanol-1 (J). 1,2-benzene dicarboxylic acid, diisooctylester (K), 9,10-secocholesta-5,7,10(19) -triene-3,24,25-triol (L), 1-Heptatriacotanol (M), 9,12,15-Octadeca Trienoic acid,2-(trimethylsilyl)oxy)-1-(trimethylsilyl)oxy) methyl)ethylester (N), 1-Nitro-2-acetamido-1,2-dideoxy-d-monnitol (O), Imidazole-2-amino-5-(2-carbxy)vinyl- (P), 2-methyl-Oct-2-enedial (Q), 1,2–15,16-Diepoxyhexadecane (R), α-D-lucopyranoside O-α–D-glucopyranosyl-(1fwdarw3) -β-D-fructofuranosyl (S), Acetic acid-1,4-dioxa spiro(4,6) undec-6-yl-ester (T), 7-Oxo-2-0xa-7-thiatricyclo(4.4.0.0(3,8) decan-4-ol (U), 2H-pyran-2-one, 5-ethylidenetetrahydro-4-(2-hydroxyethyl-) (V), 7,9-Di-tert-butyl-1-oxaspiro(4,5)deca-6,9-dione-2,8-dione (W), and betulinic acid (X).

**Table 2:** GC–MS analysis of AS3 (sub-fraction C_1_)

S/No	Name of compounds	Molecular formula	Molecular weight (g/mol.)	Retention time (minutes)
1	Spiro(androst-5-ene-17,1'-cyclobutan)-2'-one,3-hydroxy	C_22_H_32_O_2_	328	3.579
2	Propanal-2,3-dihydroxy	C_3_H_6_O_3_	90	7.112
3	3-O-methyl-d-glucose	C_7_H_14_O_6_	194	32.211
4	1,2-Benzene Dicarboxylic acid, diisooctylester	C_24_H_38_O_4_	390	63.381
5	9,10-Secocholesta-5,7,10(19)-triene-3,24,25-triol	C_27_H_44_O_3_	416	70.471
6	Urs-12-en-28-oic,3-hydroxy-methylester (ursolic acid)	C_31_H_50_O_3_	470	83.084
7	Betulinic acid	C_30_H_48_O_3_	456	85.354

**Table 3:** GC – MS analysis of AS 8 (sub-fraction C_5_)

S/No	Name of compounds	Molecular formula	Molecular weight (g/mol.)	Retention time (minutes)
1	Spiro(androst-5-ene-17,1'-cyclobutan)-2'-one,3-hydroxy	C_22_H_32_O_2_	328	3.576
2	1,4,4,7a-Tetramethyl-2,4,5,6,7,7a-hexahydro-1H-indene-1,7-diol	C_13_H_22_O_2_	210	22.238
3	Phenol,2,4-bis(1,1-dimethyl)-	C_14_H_22_O	206	29.642
4	7,9-Di-tert-butyl-1-oxaspiro(4,5) deca-6,9-diene-2,8-dione	C_17_H_24_O_3_	276	38.302
5	n-heptadecanol-1	C_17_H_36_O	256	44.966
6	1,2-Benzene dicarboxylic acid, diisooctylester	C_24_H_38_O_4_	390	63.374
7	9,10-Secocholesta-5,7,10(19)-triene-3,24,25-triol	C_27_H_44_O_3_	416	70.471
8	1-Heptatriacotanol	C_37_H_76_O	536	73.937
9	9,12,15-Octadeca Trienoic acid,2-(trimethylsilyl)oxy)-1-(trimethylsilyl)oxy) methyl)ethylester	C_27_H_52_O_4_Si_2_	496	79.882
10	Benzene propanoic acid,3,5-bis(1,1-dimethylethyl)-4-hydroxy-octadecylester	C_35_H_32_0_3_	530	83.127

**Table 4:** GC-MS analysis of AS11 (sub-fraction F)

S/no	Names of compounds	Molecular formula	Molecular weight (g/mol.)	Retention time (minutes)
1	1-Nitro-2-acetamido-1,2-dideoxy-d-monnitol	C_8_H_16_N_2_O_7_	252	3.579
2	Imidazole-2-amino-5-(2-carbxy)vinyl-	C_6_H_7_O_2_	153	24.024
3	2-methyl-Oct-2-enedial	C_9_H_14_O_2_	154	27.285
4	1,2–15,16-Diepoxyhexadecane	C_16_H_30_O_2_	254	28.431
5	α-D-lucopyranoside, O-α–D-glucopyranosyl-(1fwdarw3)-β-D-fructofuranosyl	C_18_H_32_O_16_	504	29.157
6	Acetic acid-1,4-dioxa spiro(4,6)undec-6-yl-ester	C_11_H_18_O_4_	214	31.394
7	7-Oxo-2-0xa-7-thiatricyclo(4.4.0.0(3,8)decan-4-ol	C_8_H_12_O_3_S	741	31.699
8	2H- Pyran-2-one, 5-ethylidenetetrahydro-4-(2-hydroxyethyl-	C_9_H_14_O_3_	170	32.627
9	7,9-Di-tert-butyl-1-oxaspiro(4,5)deca-6,9-dione-2,8-dione	C_17_H_24_O_3_	276	38.300
10	1,2-Benzene dicarboxylic acid, diisooctylester	C_24_H_38_0_4_	390	63.382
11	9,10-Secocholesta-5,7,10(19)-triene-3,24,25-triol	C_27_H_44_O_3_	416	70.465
12	Benzene propanoic acid,3,5-bis(1,1-dimethyl)-4-hydroxy-octadecrylester	C_35_H_62_O_3_	530	83.125

## Discussion

The calculated median LD_50_ for the plant extract was 1500.19 mg/kg of mouse body weight. This value represents the geometric mean of the highest administered dosage that resulted in no mouse fatalities. This median lethality points to the potential for inducing minimal toxicity across multiple organs. As antiplasmodial microscopic assessment mainly focuses on targeting the schizont stage of the parasite, which is responsible for clinical symptoms in patients, the evaluation of schizonticidal activity provides insight into the therapeutic effectiveness of a drug. Consequently, the evaluation of schizonticidal activity in this study, which encompassed extracts, ethyl acetate, butanol, and residual (aqueous) fractions (as presented in Table 1), revealed substantial reductions in parasite densities and improvements in mean survival time (MST) when compared to the negative control group. These findings aligned with similar research outcomes, such as a 98.63% reduction for an herbal formulation of *Mangifera indica* [15], a 75.4% reduction for the aqueous extract of *N. latifolia* stem bark [16], and 81–91% reductions for *N. pobeguini* extract [17]. Therefore, betulinic and ursolic acids alone or in combination with other bioactive components may be responsible for the plant’s root’s confirmed schizonticidal activity through binding with PfEMP-1 and PfPKG proteins in *P. berghei,* thereby leading to parasitemia reduction, as Innocente et al. [18] and de Sa et al. [19] had earlier reported that betulinic and ursolic acids and their derivatives are candidates for the development of new antimalarial drugs.

Molecular docking is the process of predicting the preferred orientation of a drug molecule when bound to a target protein. It is used for the identification of potential drug targets, the prediction of toxicity or adverse effects, as well as the design of new molecules with desired properties. Its result can be explained by looking at the energies of each binding configuration. The highest binding affinity is assumed to be the most stable binding mode, which explains why that particular molecule has the properties it does when bound to a target protein. Additionally, the binding interactions between the drug and the protein can be used to help explain the results. In this study, twenty-five (25) bioactive compounds (A–X) (Fig. 1 and Tables 2, 3 and 4) from *Nauclea latifolia* roots were selected for docking against two target proteins implicated in the pathogenesis of malaria: *Plasmodium falciparum* erythrocyte membrane protein 1 (PfEMP-1) and *Plasmodium falciparum* cGMP-dependent protein kinase (PfPKG) and compared with chloroquine drugs. Moreover, some researchers had earlier reported the biological activities of some of the characterized compounds as follows: betulinic acid has reported antiplasmodial activity [5], ursolic acids have anti-inflammatory, antioxidant, antiplasmodial, antidiabetic, and anticancer activities [10], 3-methyl glucose can inhibit the uptake of glucose in *Plasmodium falciparum *[20]*, and* 1,2-benzene dicarboxylic acid diisoctylester has been reported for its antioxidant scavenging activity towards DPPH [21].

**Fig. 2:**
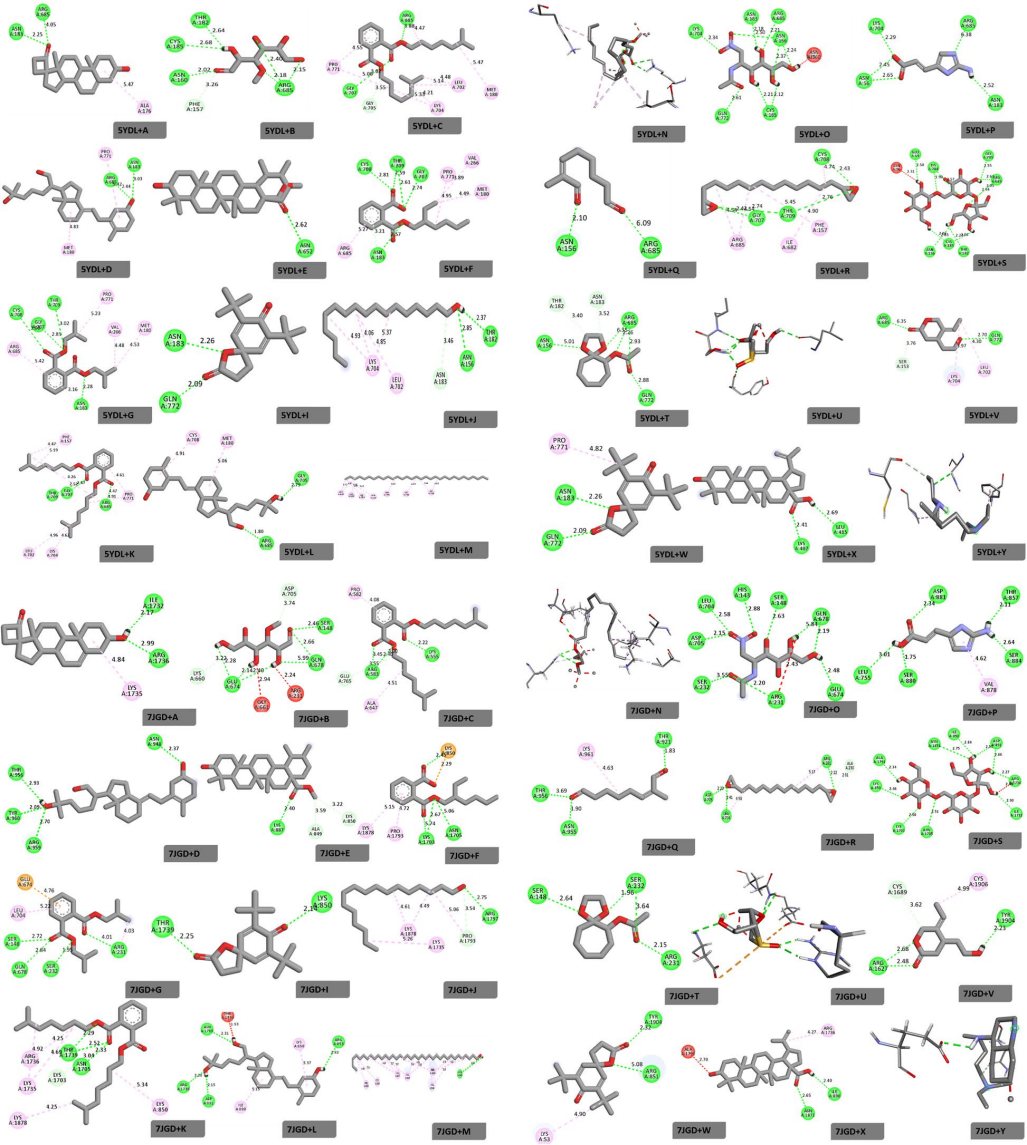
2D interactions of bioactive compounds against 5DYL and 7JGD

The results for docking scores presented in Table 5 and Fig. 2 revealed their protein–ligand interactions. According to the tabulated results for PfPKG protein (Table 5), Spiro (androst-5-ene-17,1-cyclobutan)-2-one,3-hydroxy docked with a binding energy of -7.6 kcal/mol, which was higher than those observed for 3-O-methyl-d-glucose (-4.8 kcal/mol) and Imidazole-2-amino-5-(2-carbxy)vinyl (-4.9 kcal/mol). Also, a comparative binding affinity was observed for 7,9-Di-tert-butyl-1-oxaspiro(4,5) deca-6,9-diene-2,8-dione (-8.3 kcal) and n-heptadecanol-1 (8.4 kcal/mol). Interestingly, betulinic acid recorded the highest binding affinity of -9.1 kcal/mol in comparison with chloroquine (-5.7 kcal/mol) and other bioactive compounds in the study. However, 1-Heptatriacotanol (-3.1 kcal/mol) was observed to have the lowest binding affinity and had no interactions with amino acid residues in the target protein. Moreover, the docking process with PfEMP-1 protein (Table 5) revealed binding energies of -5.8 kcal/mol and -5.9 kcal/mol for 1,2-benzene dicarboxylic acid, mono(2-ethyl hexyl)ester, and 1,2-benzene dicarboxylic acid, bis(2-methyl propyl)ester, respectively; these were higher than those found for 3-O-methyl-d-glucose (-4.9 kcal/mol) and 1,2-benzene dicarboxylic acid, diisooctylester (-5.1 kcal/mol). Betulinic acid, with a binding affinity of -8.6 kcal/mol, was higher than chloroquine (-5.7 kcal/mol) and other compounds. Nevertheless, ursolic acid (-9.7 kcal/mol) was observed to be higher than that of both betulinic acid and chloroquine. Hence, the high binding energies for these compounds imply that they are good lead compounds against the selected malaria target proteins (PfEMP-1 and PfPKG), as well as being good options for target optimization and clinical validation.

**Table 5:** Binding scores, hydrogen bonds, and bond distance for studied compounds against *Plasmodium falciparum* proteins 5DYL and 7JGD

Interaction	Binding affinity (kcal/mol)	Hydrogen bond	Residue	Bond distance
5DYL + A	-7.6	2	A:ASN:183	2.25
			A:ARG:685	4.05
B	-4.8	6	A:THR:182	2.64
			A:CYS:185	2.68
			A:ASN:160	2.02
			A:ARG:685	2.18
			A:ARG:685	2.15
			A:ARG:685	2.40
C	-6.9	2	A:GLY:707	3.02
			A:ARG:685	3.88
D	-7.3	2	A:ARG:685	2.44
			A:ASN:183	3.03
E	-9.0	1	A:ASN:652	2.62
F	-6.7	5	A:ASN:183	2.57
			A:CYS:708	2.81
			A:THR:709	2.59
			A:GLY:707	2.74
			A:THR:709	2.61
G	-6.8	4	A:CYS:708	3.06
			A:GLY:707	2.85
			A:THR:709	3.02
			A:ASN:183	2.28
I	-8.3	2	A:ASN:183	2.26
			A:GLN:772	2.09
J	-4.1	2	A:ASN:156	2.85
			A:THR:182	2.37
K	-6.7	3	A:THR:709	2.57
			A:GLY:707	2.47
			A:ARG:685	4.91
L	-7.1	2	A:ARG:685	1.80
			A:GLY:705	2.79
M	-3.1			
N	-5.4	2	A:LYS:786	2.38
			A:LYS:786	2.74
O	-5.3	9	A:LYS:704	2.34
			A:ASN:183	2.18
			A:ARG:685	2.21
			A:ASN:156	2.37
			A:ASN:156	2.24
			A:CYS:185	2.21
			A:CYS:185	2.12
			A:ASN:156	2.50
			A:GLN:772	2.61
P	-4.9	5	A:LYS:704	2.29
			A:ASN:56	2.45
			A:ASN:56	2.65
			A:ARG:685	6.38
			A:ASN:183	2.52
Q	-3.9	2	A:ASN:156	2.10
			A:ARG:685	6.09
R	-4.6	4	A:GLY:707	4.54
			A:THR:709	2.76
			A:CYS:708	4.74
			A:THR:709	4.54
S	-8.4	10	A:GLU:59	2.50
			A:LYS:704	1.90
			A:LYS:704	2.57
			A:GLY:705	2.55
			A:ARG:685	2.63
			A:ARG:685	2.05
			A:ARG:685	2.54
			A:THR:182	2.24
			A:CYS:185	2.02
			A:ASN:156	2.83
T	-5.6	5	A:ASN:156	5.01
			A:ARG:685	6.35
			A:ARG:685	2.26
			A:ARG:685	2.93
			A:GLN:772	2.88
U	-5.4	5	A:SER 75	2.00
			A:SER:75	2.02
			A:ASP:75	2.78
V	-4.9	2	A:ARG:685	6.35
			A:GLN:772	2.70
W	-8.3	2	A:GLN:772	2.09
			A:ASN:183	2.26
X	-9.1	2	A:LYS:407	2.41
			A:LEU:415	2.69
Y	-5.7	1	A:ASN:183	2.91
7JGD + A	-8.6	2	A:ILE:1732	2.17
			A:ARG:1736	2.99
B	-4.9	6	A:GLU:674	2.14
			A:GLU:674	2.28
			A:GLU:674	3.22
			A:GLU:678	5.99
			A:GLU:678	2.66
			A:SER:148	2.46
C	-5.1	3	A:ARG:583	3.45
			A:ARG:583	2.40
			A:LYS:555	2.22
D	-7.9	4	A:THR:956	2.93
			A:TYR:960	2.09
			A:ARG:959	2.70
			A:ASN:948	2.37
E	-9.7	1	A:LYS:887	2.40
F	-5.8	4	A:LYS:1703	5.74
			A:LYS:1703	2.67
			A:ASN:1705	5.06
			A:LYS:850	2.45
G	-5.9	4	A:SER:148	2.72
			A:GLN:678	2.84
			A:SER:232	1.95
			A:ARG:231	4.01
I	-6.8		A:THR:1739	2.25
			A:LYS:850	2.14
J	-3.9	1	A:ARG:1767	2.75
K	-5.8	3	A:THR:1739	2.29
			A:THR:1739	2.52
			A:ASN:1705	2.33
			A:ASN:1705	3.04
L	-7.0	4	A:ARG:1736	2.20
			A:ASP:891	2.15
			A:ASN:1705	2.31
			A:ARG:853	2.92
M	-4.3	1	A:ASN:948	2.63
N	-5.2	3	A:ASN:1705	2.85
			A:ASN:1705	2.37
			A:THR:1739	1.93
O	-5.9	9	A:ASP:705	2.15
			A:LEU:704	2.58
			A:HIS:143	2.88
			A:SER:148	2.63
			A:GLN:678	5.84
			A:GLN:678	2.19
			A:GLN:674	2.48
			A:ARG:231	2.20
			A:SER:3.55	3.55
P	-5.1	5	A:LEU:755	3.01
			A:SER:880	1.75
			A:ASP:881	2.34
			A:THR:857	2.11
			A:SER:884	2.64
Q	-4.5	3	A:ASP:955	1.90
			A:THR:956	3.69
			A:THR:921	1.83
R	-4.2	3	A:ASP:705	2.20
			A:LEU:704	2.41
			A:ARG:231	2.22
S	-8.7	10	A:ALA:1791	2.34
			A:LYS:850	2.46
			A:LYS:1703	2.50
			A:ASN:1705	2.51
			A:ILE:1732	2.90
			A:ARG:1736	2.27
			A:ASP:891	2.53
			A:ILE:890	2.84
			A:ASN:1871	2.75
			A:ALA:1791	2.34
T	-5.8	4	A:SER:148	2.64
			A:SER:232	1.96
			A:SER:232	3.64
			A:ARG:231	2.15
U	-5.5	5	A:ARG:231	2.10
			A:GLN:678	2.62
			A:GLN:678	2.82
			A:ASP:705	2.80
			A:ARG:231	2.10
V	-6.4	3	A:ARG:1627	2.68
			A:ARG:1627	2.48
			A:TYR:1904	2.23
W	-6.7	2	A:TYR:1904	2.32
			A:ARG:851	5.08
X	-8.6	2	A:ASN:1871	2.65
			A:ILE:890	2.40
Y	-5.7	1	A:ASP:891	2.81

Furthermore, adsorption, distribution, metabolism, excretion, and toxicity (ADMET), as critical pharmacokinetic parameters, play a central role in the development of new drugs. A quality drug should possess good ADMET properties at a therapeutic dose. Recently, many in silico models have been developed for the estimation of chemical ADMET properties; therefore, ADMET properties are paramount in all phases of the drug development process. In this study (Table 6), α-D-glucopyranoside O-α-D-glucopyranosyl-(1fwdarw3)-β-D-fructofuranosyl with molecular weight (504.44 g/mol) and hydrogen bond acceptor (16) was higher than those of ursolic acid (472.74 g/mol) and betulinic acid (456.70 g/mol). The greater the molecular mass of a compound, the likelier its non-drug-likeness, as its absorption in the GIT might be affected. The low GIT absorption recorded for the three compounds might be related to their high molecular weights; hence, their reduced bioavailability and inability to cross the blood–brain barrier. However, for drug-likeness, betulinic and ursolic acids revealed better results as compared to α-D-glucopyranoside O-α-D-glucopyranosyl-(1fwdarw3)-β-D-fructofuranosyl because of their lower molecular weights, non-permeation of the blood–brain barrier, non-inhibition of metabolizing enzymes, and non-violation of Lipinski’s rules. In addition, both betulinic and ursolic acids had better bioavailability values, whereas α-D-glucopyranoside O-α-D-glucopyranosyl-(1fwdarw3)-β-D-fructofuranosyl had the highest synthetic accessibility value, implying its prospect of generating different synthetic ligands. The indices of the *P* values suggested that the ADMET data for the three compounds showed no significant difference; therefore, the presented data are quite critical in the choice of validating both ursolic and betulinic acids as drug candidates from *Nauclea latifolia* roots for the treatment of malaria.

**Table 6:** ADMET properties of the best-performed bioactive compounds

	Urs-12-en-28-oic,3-hydroxy-methylester (E)	α-D-glucopyranoside O-α–D-glucopyranosyl-(1fwdarw3)-β-D-fructofuranosyl (S)	betulinic acid (X)
**Physicochemical properties**			
Formula	C_31_H_50_O_3_	C_18_H_32_O_16_	C_30_H_48_O_3_
Molecular weight	472.74 g/mol	504.44 g/mol	456.70 g/mol
No.of heavy atoms	34	34	33
No. of rotatable bonds	2	8	2
H.bonds acceptor	3	16	3
H.bond donor	1	11	2
TPSA	46.53 Å^2^	268.68 Å^2^	57.53 Å^2^
**Pharmacokinetics**			
GI absorption	Low	Low	Low
BBB permeant	No	No	No
P-gp substrate	No	Yes	No
CYP2C19 inhibitor	No	No	No
CYP2C9 inhibitor	No	No	Yes
CYP2D6 inhibitor	No	No	No
CYP3A4 inhibitor	No	No	No
Log *K*_p_ (skin permeation)	-3.08	-13.53 cm/s	-3.26 cm/s
**Druglikeness**			
Lipinski	Yes	No	Yes
Ghose	No	No	No
Veber	Yes	No	Yes
Muegge	No	No	No
Bioavailability score	0.55	0.17	0.85
Synthetic accessibility	5.54	6.28	5.63
**Lipophilicity**			
Log *P*_o/w_ (iLOGP)	4.64	1.41	3.83
Log *P*_o/w_ (XLOGP3)	8.60	-5.85	8.21
Log *P*_o/w_ (WLOGP)	7.26	-7.57	7.09
Log *P*_o/w_ (MLOGP)	6.12	-6.56	5.82
Log *P*_o/w_ (SILICOS-IT)	6.02	-5.93	5.75
Consensus Log *P*_o/w_	6.53	-4.90	6.14

## Conclusion

The findings from this study led to the conclusion that the residual fraction from *Nauclea latifolia* roots has the best therapeutic index against *Plasmodium berghei* and that twenty-five (25) bioactive compounds were characterized from the plant. However, the docking of the compounds with PfEMP-1 and PfPKG generated their binding affinities, bond distances, hydrogen bonds, and amino acid residues. Betulinic and ursolic acids were better than other compounds, including chloroquine, in terms of their binding affinities, molecular weight, non-permeation of the blood–brain barrier, non-inhibition of metabolizing enzymes, and non-violation of Lipinski’s rules. Therefore, the lead compounds are candidates for lead optimization and further rational drug design processes as observed against *Plasmodium falciparum* target proteins (PfEMP-1 and PfPKG) that are implicated in malaria pathogenesis.

## Data Availability

All datasets generated in this study are available from the corresponding author on request.
